# A Case of Revelation Due to Pegfilgrastim

**DOI:** 10.7759/cureus.63777

**Published:** 2024-07-03

**Authors:** Kevin T Dao, Kishan Ghadiya, Elias Inga Jaco, Rupam Sharma, Roshun Rahimi, Marah Sukkar, Moujin Adebayo, Janpreet Bhandohal, Harmanjeet Dhillon, Fowrooz Joolhar

**Affiliations:** 1 Internal Medicine, Kern Medical, Bakersfield, USA; 2 Cardiology, Kern Medical, Bakersfield, USA

**Keywords:** medication induced, multidisciplinary, granulocyte colony-stimulating factor, jaw mass, neulasta, pegfilgrastim

## Abstract

Pegfilgrastim is a granulocyte colony-stimulating factor used in non-myeloid cancer patients to prevent infections and neutropenic fevers. Although this medication is widely used to induce granulocytosis in pancytopenia patients, there are certain instances where such a situation can cause severe side effects. In this case, we present a patient with a history of metastatic colon cancer who is currently taking pegfilgrastim to counter the agranulocytosis caused by his chemotherapy treatment. However, the patient shortly developed localized left-sided jaw swelling, and upon further investigation, the granulocyte colony-stimulating factor revealed an underlying bacteremia. A discussion will also be held regarding the mechanism of action of how pegfilgrastim induced this patient’s symptoms as well as the risks and benefits.

## Introduction

In many instances, pegfilgrastim is a very effective medication to circumvent certain side effects of chemotherapy regimens, which can make patients quite susceptible to infections. Therefore, studies have shown the safety and efficacy of granulocyte colony-stimulating factors, particularly pegfilgrastim, as a regimen to prevent febrile neutropenia and infections as well as other chemotherapy-related complications [1-3]. Unfortunately, like many other medications, both intentional and unintentional side effects can occur. Commonly, patients with cancer tend to have complex pathologies that can involve multiple organ systems. In these situations, multidisciplinary healthcare management is needed to ensure appropriate care. However, there can be some forms of management from an oncological standpoint that might not be ideal from a cardiological point of view, and vice versa. Nonetheless, questions among specialties should be raised to ensure patient care. Here, we would like to go into further detail as to how pegfilgrastim was able to reveal an underlying disease that wouldn’t have been discovered due to the patient’s immunocompromised state. A conversation as to the risk of using this medication in this patient will also be discussed.

## Case presentation

A 68-year-old male with a significant history of metastatic colon cancer, aortic valve replacement requiring anticoagulation, pacemaker placement secondary to bradycardia, and aortic aneurysm status post-stent placement presented to the emergency department (ED) due to left facial swelling with tenderness after recent use of pegfilgrastim. The patient noted that he was initially diagnosed with grade 2 mucinous adenocarcinoma with positive lymph nodes a few years ago, requiring a hemicolectomy and biopsy. He was then treated with capecitabine and oxaliplatin, which unfortunately failed, requiring the patient to switch chemotherapy treatment to bevacizumab and 5-fluorouracil with leucovorin. However, he developed severe neutropenia and, as such, was prescribed pegfilgrastim by his oncologist. Initially, after receiving one dose, the patient noted a small bump on his left parotid gland and ignored it since he had no other symptoms. A few weeks later, the patient received a second dose of pegfilgrastim, noted severe swelling of his jaw, and decided to present to the ED.

**Figure 1:**
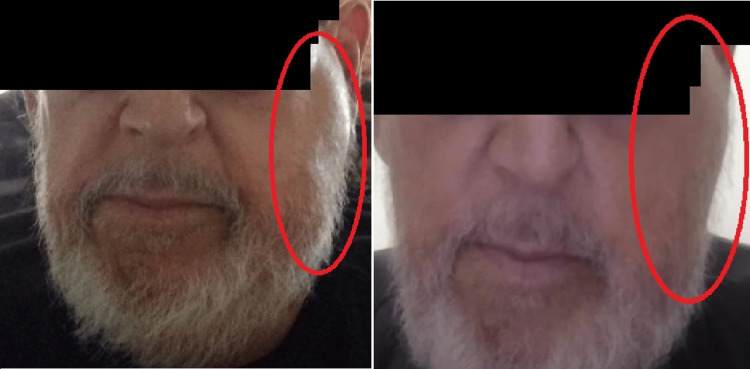
Left-sided mandibular swelling upon admission The red circle denotes the swelling of the patient's mandible.

At presentation, the patient’s vitals were unremarkable, and the physical exam showed no pockets of purulence or fluctuance of the left mandible but tenderness to palpation of the area (Figure 1). All other physical exam findings were unremarkable. A complete blood count, basic metabolic panel (Table 1), and blood cultures were taken with the cessation of pegfilgrastim and the initiation of ceftriaxone and dexamethasone.

**Table 1:** Pertinent basic metabolic panel and blood count

Laboratory Values	Reference Range	Admission	Discharge
Procalcitonin	<0.5 ng/mL	5.16 ng/mL	N/A
Blood urea nitrogen	7-18 mg/dL	28 mg/dL	16 mg/dL
Creatinine	0.67 – 1.17 mg/dL	0.66 mg/dL	0.71 mg/dL
Alkaline phosphatase	45-117 unit/L	101 unit/L	78 Unit/L
Alanine transaminase (ALT)	13-61 unit/L	12 unit/L	10 unit/L
Aspartate aminotransferase (AST)	15-37 unit/L	22 unit/L	17 unit/L
Lactic acid	0.4 – 2 mmol/L	1.3 mmol/L	N/A
Prothrombin time	12.5 – 14.3 Sec	41 Sec	14.7 Sec
Partial thromboplastin time	25.4 – 37.6 Sec	94.5 Sec	53.9 Sec
White blood count	4.5 - 11 x10^/mcl	17.9 x 10^3/mcl	2.1 x 10^3/mcl
Red blood count	4.26 – 5.8 x10^6/mcl	3.73 x 10^6/mcl	3.86 x 10^6/mcl
Hemoglobin	13.2 – 17.4 g/dL	11 mg/dL	10. 8 g/dL
Hematocrit	38.9 – 51 %	32 %	33 %
Mean corpuscular volume	80-98	85.8	85.7
Platelets	150 - 450 x 10^/mcl	67 x 10^3/mcl	136 x 10^3/mcl
Neutrophil %	50-70%	97.2 %	65.1 %
Lymphocyte %	20-45 %	2 %	16.4 %
Bands %	<12%	0 %	4 %

**Figure 2:**
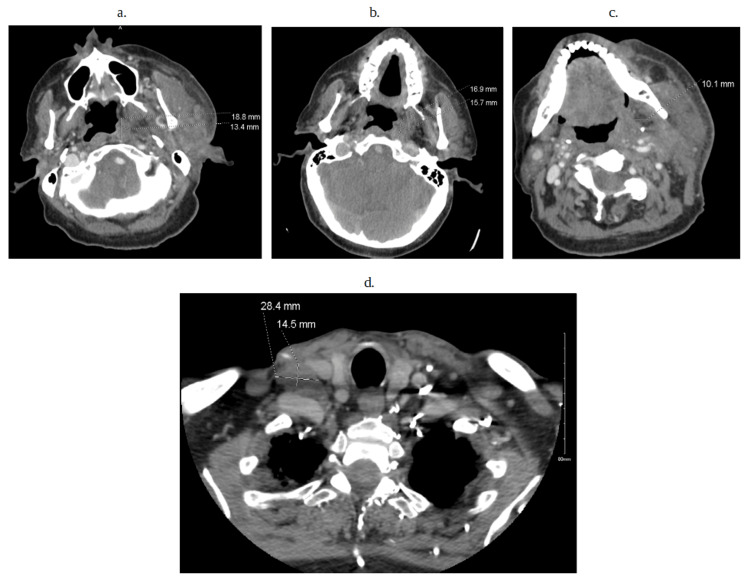
Computed tomography of the head and neck Figure 2a: shows an 18.8 x 13.4 mm nodule of the face on the left side of the fossa of Rosenmuller. The possibility of metastatic disease should be considered, given the possibility that a second primary left neck mass can’t be excluded. Figure 2b: shows a 16.9 x 15.7 mm enlarged lymph node. Figure 2c: shows that there is no evidence of enhanced encapsulated collection to suggest a frank abscess, but there is a 10 mm low attenuation collection in the left submandibular space that could represent a small phlegmon. Figure 2d: shows a 28 x 14 mm right superclavicular lymph node.

**Figure 3:**
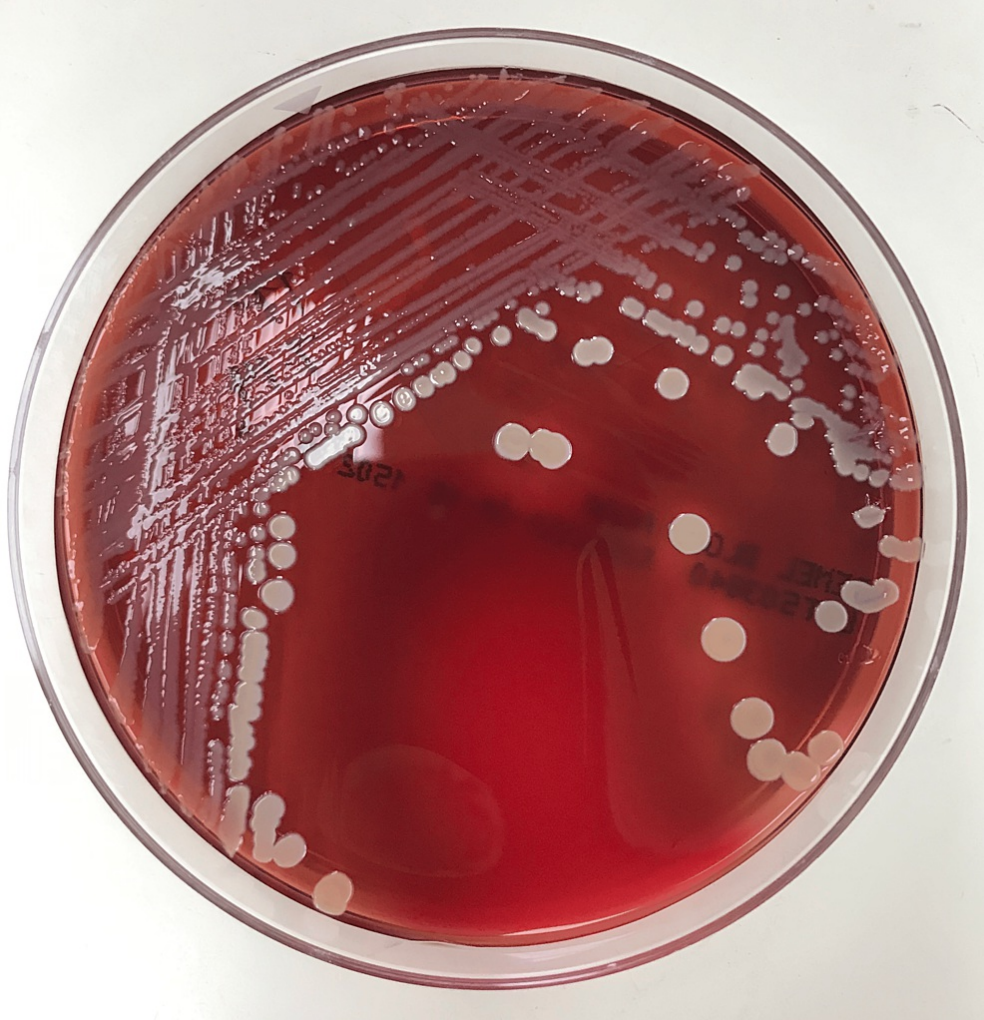
Patient’s blood culture that was noted to grow methicillin susceptible Staphylococcus aureus

**Figure 4:**
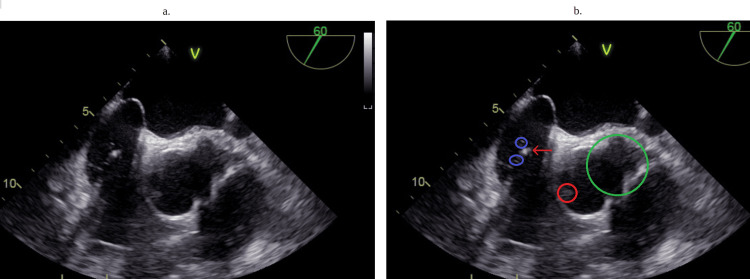
Transesophageal echocardiogram Figure 4a: shows an unmarked image of the patient's transesophageal echocardiogram. Figure 4b: shows mobile structures that are attached to leads suggestive of various vegetation along the leads of the pacemaker, which are circled in blue, while the red arrow is one of the pacemaker leads. The red circle marks the vegetation that is noted on the aortic valve, and the green circle is where an aortic aneurysm is present.

Computed tomography of the head and neck (Figure 2a-2d) was remarkable for various lymph node enlargements on both sides of the neck without exclusion of metastasis. Blood cultures obtained grew methicillin-sensitive Staphylococcus aureus (Figure 3). The transthoracic echocardiogram demonstrated aortic valve regurgitation, but no vegetation was seen initially. However, due to the patient's cardiac hardware and port-a-cath placement, a transesophageal echocardiogram (TEE) (Figure 4a, 4b) was done. TEE findings were suspicious for a small-sized patent foramen ovale (PFO). Multiple echogenic mobile structures attached to the catheter tip and pacemaker wiring were suggestive of numerous vegetations arising from the cardiac apparatus. A small vegetation was also appreciated at the base of the mechanical aortic valve rim (Figure 4a, 4b), consistent with damaged and infiltrated valvular disease. The mechanical valve was considered to be functioning appropriately with no regurgitation.

An interdisciplinary team meeting was then held between infectious disease, oncology, internal medicine, cardiology, and interventional radiology for further consideration of treatment plans. Discussions revolved around an aggressive approach with surgery to remove the mechanical valve versus a conservative approach via medicine while considering his prognosis secondary to metastatic colon cancer. Questions regarding whether a patient was even a reliable surgical candidate were also heavily considered. After multiple meetings with many medical teams as well as with the patient, a conservative medical approach was deemed a better course of action.

**Figure 5:**
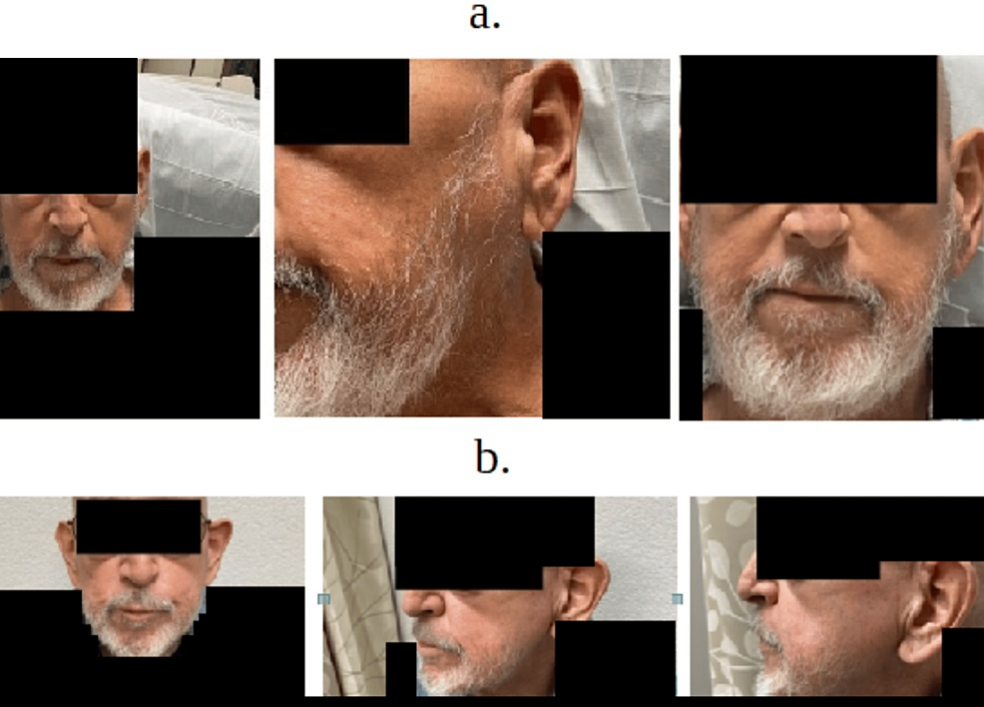
Left-sided mandibular after treatment and follow-up Figure 5a: left-sided mandibular after treatment; Figure 5b: left-sided mandibular on outpatient follow-up appointment

The patient was initially started on ceftriaxone 1 gram every 24 hours and vancomycin 1.5 gram twice daily the first few days, but was transitioned to cefazolin 2 gram IV three times a day for 42 days, along with gentamicin 3 mg/kg IV daily for 14 days after sensitivities. Nine days after hospitalization, the patient began to improve clinically, with the greatest improvement in his toleration of a regular oral diet and facial mass (Figure 5a). Blood cultures were negative after antibiotics. He remained hemodynamically stable while admitted to the ward and without any spikes in temperature. The patient was discharged on IV cefazolin 2 grams for eight hours for 42 days and oral rifampin 500 milligrams daily for one month. He continued to follow up with radiology-oncology for possible radiotherapy status post-discharge, as well as with oncology, infectious disease, cardiology, and internal medicine teams for status post-hospitalization discharge for management of IV antibiotic outpatients.

At his two-month follow-up, the patient stated that he was feeling well and that his left-sided facial swelling had remarkably decreased. He endorses that no swelling has occurred after stopping pegfilgrastim treatments and that he has had no active complaints. The patient also noted that he has various oncology appointments for further discussions regarding management for his metastatic cancer (Figure 5b).

## Discussion

Pegfilgrastim is a long-acting form of filgrastim, a granulocyte-stimulating factor designed to increase the body’s neutrophil level by inducing proliferation, differentiation, and maturation of hematopoietic stem cells [1,2]. Treatment is not usually considered unless patients have significant risk factors that make them susceptible to infection [4]. For patients who have non-myeloid malignancies and are receiving a high-risk chemotherapy regimen, pegfligrastim can be heavily considered, despite the side effects [5]. Some case reports have noted mild symptoms in breast cancer patients, such as fever, diarrhea, and nausea, to more severe symptoms such as renal insufficiency and bone pain [6,7]. In some instances, there have been other unique cases with more complicated side effects, such as large cell vasculitis, particularly in the aorta and subclavian arteries [8,9]. Despite this, there are very few instances where pegfligrastim has induced jaw swelling or lymph node enlargement. Although one very important side effect that is pertinent is that there has been documentation of pegfligrastim causing excessive hyperleukocytosis [10]. In said case report, it has been shown that the medication has the potential to increase the white blood count to 149 x 10^3/microL and the absolute neutrophil count to 110 x 10^3/microL [10]. As such, further details regarding the mechanism by which this medication caused jaw swelling and how it relates to this case will be discussed in depth.

Due to the patient’s history of metastatic colon cancer, it was first hypothesized that pegfilgrastim caused neutrophilic proliferation and infiltration of areas where cancer cells were present [1-2,10]. This hypothesis was supported by the fact that the patient only noted a small mass on his left parotid glands after his first dose. Despite this, the possibility of an infection-related cause was still very possible due to the patient's longstanding immunocompromised state and history of cardiac hardware [11].

CT of the neck and head (Figure 2a-2d) showed various enlarged nodules in multiple bilateral areas of the neck. It was believed that due to the patient's valvular vegetation (Figure 4a, 4b) and positive blood cultures (Figure 3), there was suspicion of a possible septic emboli that was exported to the jaw. Yet, a physical exam showed no pockets of purulence despite being tender to palpation. A CT scan also showed no evidence of abscess or pockets of necrosis, but the possibility of a small phlegmon was present (Figure 2c), with the concept of metastatic disease also possible (Figure 2a). Unfortunately, a metastatic cause would be difficult to prove since a biopsy or surgical intervention of an enlarged lymph node had to be done to rule in the presence of cancer cells, and such a procedure would not have changed the patient's course of treatment. What is clear is that the root of the patient’s jaw swelling is due to the use of pegfilgrastim, which helped reveal an underlying infection that the patient’s immunocompromised body could not mount a response to. Fortunately, the patient had not developed an inappropriate physiological response, or septic shock, after receiving his dose of pegfilgrastim.

As a result, to prevent the patient from going into shock, it was necessary to cease the medication and begin steroids. Albeit, the question still remains on how to treat this patient moving forward. In this case, it was decided that treating this patient's infection while ensuring hemodynamic stability was the highest priority [12,13]. Therefore, the oncologist agreed to temporarily halt the patient’s chemotherapy treatments. Infectious disease physicians were then recommended to continue monitoring blood cultures with empiric antibiotics. Our cardiologist and interventional radiologist also discussed medical management vs. replacement of the patient's infected valves, pacemaker, and implantable cardioverter defibrillator (ICDs) (Figure 4a, 4b) [14,15]. Unfortunately, in many cases, using antibiotic treatment to manage infected hardware hasn’t been done but rather just as a prophylactic measure [16-18]. Most surgical options were abandoned when the National Surgical Quality Improvement Program (NSQIP) criteria showed that the patient was a poor surgical candidate. A conversation was also held with the patient’s preferred approach, and it was concluded that medical management was ideal despite reaching an impasse about the use of antibiotic treatment for infected hardware rather than removing it. The patient knew the unfavorable risk of surgery and ultimately agreed to continue with outpatient cefazolin 2 g q8 and 900 mg rifampin PO daily x 5 with close follow-up and was discharged.

## Conclusions

Overall, pegfilgrastim is a very unique drug, and despite its effects on increasing granulocytes, physicians should be aware of the unintended consequences that can occur with such an effect. This case raises the possibility that, when starting such a medication, there should be caution that patients should be screened for any underlining infections to prevent complications, particularly septic shock. This is particularly true for patients who have certain risk factors, such as hardware, that make them more susceptible. Fortunately, the patient's neutrophilia resulted in left-sided jaw swelling, which was a mild consequence when compared to the lethal complications, such as septic shock, that could have arisen. Although this can be done on a case-by-case basis, hopefully complicated case reports like this can help physicians be alert to the overall consequences of drug effects and help prevent fatal complications.

## References

[1] Bedell C (2003). Pegfilgrastim for chemotherapy-induced neutropenia. Clin J Oncol Nurs.

[2] Yang BB, Kido A (2011). Pharmacokinetics and pharmacodynamics of pegfilgrastim. Clin Pharmacokinet.

[3] Sharma DC (2004). Pegfilgrastim lowers side-effects of chemotherapy. The Lancet Oncology.

[4] Smith TJ, Bohlke K, Lyman GH (2015). Recommendations for the use of WBC growth factors: American Society of Clinical Oncology clinical practice guideline update. J Clin Oncol.

[5] Crawford J, Moore DC, Morrison VA, Dale D (2021). Use of prophylactic pegfilgrastim for chemotherapy-induced neutropenia in the US: a review of adherence to present guidelines for usage. Cancer Treat Res Commun.

[6] Najafi S, Ansari M, Kaveh V, Haghighat S (2021). Comparing the efficacy and side-effects of PDLASTA® (Pegfilgrastim) with PDGRASTIM® (Filgrastim) in breast cancer patients: a non-inferiority randomized clinical trial. BMC Cancer.

[7] Moore DC, Pellegrino AE (2017). Pegfilgrastim-induced bone pain: a review on incidence, risk factors, and evidence-based management. Ann Pharmacother.

[8] Saito H, Suda T, Oishi N, Matsushita E (2021). Pegfilgrastim-induced large vessel vasculitis. BMJ Case Rep.

[9] Costa Silva R, Monteiro M, Dias RP (2021). Large-vessel vasculitis induced by pegfilgrastim. Acta Reumatol Port.

[10] Snyder RL, Stringham DJ (2007). Pegfilgrastim-induced hyperleukocytosis. Ann Pharmacother.

[11] Crawford J, Dale DC, Lyman GH (2004). Chemotherapy-induced neutropenia: risks, consequences, and new directions for its management. Cancer.

[12] Mirouse A, Vigneron C, Llitjos JF (2020). Sepsis and cancer: an interplay of friends and foes. Am J Respir Crit Care Med.

[13] Bou Chebl R, Safa R, Sabra M (2021). Sepsis in patients with haematological versus solid cancer: a retrospective cohort study. BMJ Open.

[14] Nagarakanti S, Bishburg E, Bapat A (2020). Cardiac implantable electronic device infection: does the device need to be extracted?. J Arrhythm.

[15] Sohail MR, Uslan DZ, Khan AH (2007). Management and outcome of permanent pacemaker and implantable cardioverter-defibrillator infections. J Am Coll Cardiol.

[16] Tarakji KG, Mittal S, Kennergren C (2019). Antibacterial envelope to prevent cardiac implantable device infection. N Engl J Med.

[17] Mounsey JP, Griffith MJ, Tynan M, Gould FK, MacDermott AF, Gold RG, Bexton RS (1994). Antibiotic prophylaxis in permanent pacemaker implantation: a prospective randomised trial. Br Heart J.

[18] Bluhm G, Jacobson B, Julander I, Levander-Lindgren M, Olin C (1984). Antibiotic prophylaxis in pacemaker surgery--a prospective study. Scand J Thorac Cardiovasc Surg.

